# Does attitude importance moderate the effects of person-first language? A registered report

**DOI:** 10.1371/journal.pone.0300879

**Published:** 2024-03-28

**Authors:** Sandy Schumann, Hazem Zohny

**Affiliations:** 1 Department of Security and Crime Science, University College London, London, United Kingdom; 2 Oxford Uehiro Centre for Practical Ethics, University of Oxford, Oxford, United Kingdom; NYU Grossman School of Medicine: New York University School of Medicine, UNITED STATES

## Abstract

Previous research has demonstrated that exposure to outgroup descriptions that use person-first, as compared to identity-first, language can attenuate negative stereotypes or prejudice and enhance support for policies that seek to advance outgroup rights. However, those benefits of person-first language may not apply to all social groups equally. The present study examines a boundary condition of the effects of person-first language. Specifically, we postulate that person-first language reduces the stigmatization of outgroups to a lesser degree if individuals hold more important negative attitudes towards the respective communities. We will test this hypothesis in a two-factorial 2 (target group) x 2 (descriptor) online experiment that includes a control group and for which we will recruit a general-population sample (*N* = 681). Stereotyping, dehumanization, as well as negative affect and behavioral intentions towards two outgroups will be compared: people with a physical disability/the physically disabled (i.e., negative attitudes are expected to be less important) and people who have committed a violent crime/violent criminals (i.e., negative attitudes are expected to be more important). Our findings will bear implications for understanding when language use could influence public opinion of different social groups. Additionally, the research can inform the development of more effective communication policies to promote inclusion and reduce stigma.

## Introduction

In recent years, there has been a surge in guidelines that encourage the adoption of inclusive, non-stigmatizing language (e.g., [1–4]. Especially noteworthy is the recommendation to use person-first (or person-centered) instead of identity-first language; doing so has been most commonly suggested in medical and criminal justice contexts [5]. In practice, when communicating with or about an individual, it is, for example, recommended to say "person with autism" and not "autistic". Or, rather than "ex-convict", the phrase "person who was formerly incarcerated" is preferred (i.e., a post-modified noun that refers to a person is followed by a descriptor).

Despite criticism of person-first language policies [6–8], empirical evidence points to its benefits. More precisely, exposure to outgroup descriptions that employ person-first language has been found to reduce negative outgroup stereotypes, prejudice, and stigma (e.g., [9–12]. Having said this, it is also evident that person-first language may not affect perceptions of and attitudes or behavior towards all social groups equally. St. Louis [13] showed that across 12 different descriptor pairings, person-first language evoked more positive impressions for only two outgroups—“persons with leprosy” and “persons with psychosis”. Additionally, effect sizes that were attained in studies that explored the influence of person-first language in medical and criminal justice contexts varied widely [11, 14]. The reasons for those differential result patterns were not examined systematically.

The present research addresses this gap in the literature. Specifically, we investigate whether attitude importance moderates the effects of person-first language such that the latter are weaker for outgroups towards which individuals hold more important negative attitudes [15, 16]. Testing this hypothesis, we will recruit a general-population sample and conduct an online experiment to compare negative and positive stereotyping, dehumanization, negative affect, and behavioral intentions directed at others who have committed a violent crime/violent criminals (i.e., negative attitudes are expected to be more important) and people with a physical disability/the physically disabled (i.e., negative attitudes are expected to be less important).

## Person-first language and its benefits

Efforts to promote person-first language [1–4], which have their origins in the disability rights movement in the 1970s, the 1980s Denver Principles, as well as prison reform and Black liberation movements in the 1960s-70s [17], aim to alleviate stigmatization and its detrimental implications on mental/physical health, access to support, and social inclusion [17, 18]. Referring, for instance, to someone as “a person with an addiction” is thought to emphasize their personhood over the illness; their “humanity (and dignity is preserved) while promoting … individuality” [7, p. 258]. Moreover, person-first language is expected to ensure that addictive behavior, in this example, is viewed as just one of many actions that shape the person’s identity [5, 19]. In avoiding essentialization, it is indicated that the stigmatized characteristic or behavior can be changed [6].

To date, empirical evidence that confirms the benefits of person-first language is limited to studies that have examined its influence on outgroup perceptions, attitudes, and behavioral intentions [20–23] (see Lynch and Groombridge [24] for nil effects and Feldman et al. [25] who demonstrated no relationship between the *use* of person-first language and attitudes towards individuals with disabilities). Ashford and colleagues [9] employed the Go/No-Go Association Task to compare implicit negative biases towards individuals described by the terms "substance abuser" or "person with substance use disorder". The authors found significantly more negative implicit outgroup attitudes associated with the label "substance abuser". Endorsing these findings, “addicts” were viewed as more inferior than “people with addiction”, and participants endorsed maintaining social distance to “addicts” more strongly; sympathy and helping intentions were also lower for “addicts” [10]. Furthermore, a study that recruited professional counselors reported that participants expressed higher levels of tolerance towards “people with mental illnesses” than “the mentally ill” [14].

Beyond the medical domain, the effects of person-first language have been documented in two studies pertaining to the criminal justice context as well [18, 26]. Reading instructions that referred to another individual as an "offender" elicited more stigma and negative judgments than instructions that made reference to a "person with a conviction" [11]. Describing individuals either as a “returning citizen”, “a person who was formerly incarcerated” (i.e., two different person-first descriptors), or an “ex-convict” (i.e., identity-first descriptor) also affected outgroup perceptions and intentions to support reintegration efforts [12]. Notably, negative stereotypes were increased, willingness for social closeness reduced, and reintegration measures were less endorsed when participants were exposed to an identity-first descriptor [12].

## Criticism of person-first language

Despite those promising findings, person-first language has also attracted criticism. First, identity-first, not person-first, language is thought to enable a higher expression of agency and autonomy, allowing individuals and communities with lived experience to claim terms like “autistic” or “disabled” [20]. Moreover, it has been argued that separating a person from, for instance, their smoking behavior could suggest that the individual is otherwise not valuable [6]. Additionally, person-first language might, in fact, highlight that certain behaviors or qualities are negative, thus promote stigmatization [20, 21]. Speaking to this point, Gernsbacher [8] showed that person-first language was “more frequently (used) to refer to children with disabilities than to refer to children without disabilities” (para. 8), and was, overall, more frequently used to denote more stigmatized characteristics.

Relatedly, person-first language might not always align with someone’s self-image [6] or how people prefer to describe themselves. A study with participants who had received an autism diagnosis [27] demonstrated that there were no differences in the preference of the terms “autistic”, “person on the autism spectrum”, or “autistic person”; however, the aforementioned three terms were more preferred than “person with autism”, “person with autism spectrum disorder”, and “person with autism spectrum condition”. Thus, person-first language was evaluated less positively than identify-first language if it specified a disorder or condition [27, 28].

## Generalizing the person-first language effect

A further concern regarding the use of person-first language that has not been explored systematically pertains to the generalizability of its benefits. In reviewing the literature, it is evident that person-first language does not affect outgroup attitudes and behavioral intentions to the same degree across different social groups. For example, the prevalence of negative implicit associations differed to a moderate extent between participants exposed to the terms "substance abuser" or "person with a substance use disorder" (d = .39; [9]). A moderate-large difference in levels of sympathy or willingness to help was documented when others were described as "addicts" instead of "people with addiction” (d = .61; [10]; see Granello and Gibbs [14] for moderate-large effects when comparing attitudes towards “people with mental illnesses” and “the mentally ill”). Exposure to person-first rather than crime-first language, in turn, led to only a small decrease in perceived recidivism risk of violent offenders (d = .21; [11]). The same study showed that person-first language did not at all impact perceptions of those who had committed nonviolent property or drug offenses ([11]; see Jackl [12] for further small effects comparing person- or crime-first language).

Similarly, St. Louis [13] demonstrated that person-first language evoked more positive outgroup impressions for only two of twelve descriptor pairing–“person with leprosy”/”leper” and “person with psychosis”/”psychotic”. These findings were attained in a diverse sample that included individuals with a language-disorder, parents of clients, speech-language pathology students, and the general public. St. Louis [13] did not elaborate on why person-first language effects were not identified for all descriptor pairings.

## Attitude importance as a moderator of the effects of person-first language

Advancing the literature, we seek to investigate one potential explanation for the differential result patterns presented in the previous section. As noted, person-first language is expected to attenuate stigmatizing views (and behavior) such that different, more humanizing outgroup attitudes (and actions) are adopted. However, exposure to outgroup descriptions that use person-first language should only influence attitudes, or influence those attitudes more substantially, that are, in fact, malleable. A burgeoning body of research confirms that the latter is not the case for all viewpoints. Specifically, there is evidence to suggest that attitudes that are *stronger* are more resistant to change [29–33].

Attitude strength is distinguished from valence. Even if two persons evaluate an object negatively (i.e., same valence), the strength of the respective negative attitudes can vary [34]. Attitude strength also varies between objects, and someone might hold stronger and weaker attitudes about different objects [16]. Furthermore, groups of people (i.e., issue publics; [35]) may be characterized by equally strong attitudes towards the same object [36], attitude strength fluctuates collectively in line with the political climate and public attention on certain issues.

A common operationalization of attitude strength is attitude importance, that is, “the subjective sense of concern, caring, and the significance that an individual attaches to an attitude (towards a given object)” ([37], p. 209). Boninger and colleagues [38] stipulated that attitude importance reflects a belief about the significance of an attitude for oneself and forms part of one’s self-concept [39]. This subjective judgment is thought to be independent of notions of descriptive or prescriptive importance (i.e., what attitudes most people attribute significance to or what is the right attitude to attribute significance to).

Three predictors of attitude importance have been identified [15]. An attitude is typically more important if the object it pertains to is of higher material self-interest (i.e., impacts the individual’s rights, privileges, or lifestyle), if the attitude (object) is of higher importance to a social group that the individual strongly identifies with, and if the attitude (object) reflects or makes reference to core moral, ideological, or aesthetic values [38]. Self-interest must be differentiated from being personally affected by a situation or condition (i.e., personal relevance/involvement) [15]. In Boninger and colleagues’ [38] seminal work it was also demonstrated that self-interest, social identification, and values influenced the importance of distinct attitudes to different degrees. On the issue of racial segregation, for example, self-interest primarily informed attitude importance; regarding the topic of abortion, self-interest and values predicted importance at comparable levels [38].

As noted earlier, a burgeoning body of research has shown that more important attitudes are more resistant to change even if the person is confronted with substantial contesting evidence [40]. In a field study that examined the impact of participating in a class on women’s studies on egalitarian attitudes, the latter were less affected if students viewed the class as more important [41]. In turn, inoculation (or forewarning) messages did not foster further resistance (i.e., predicted less attitude change) for individuals who considered their attitudes on the topic more important; forewarning did boost resistance for those who attributed low significance to the issue [42].

The persistence of more important attitudes has been primarily explained by focusing on cognitive processes. The elaboration likelihood model of attitude strength (Petty et al., 2009), for example, posits that information associated with important attitudes is processed in a more elaborate manner, which would enhance attitude accessibility and certainty, as well as knowledge about the attitude object. Indeed, important attitudes are typically more salient [32, 43]. However, affect appears to play a crucial role as well in maintaining more important attitudes. If an important attitude was contested, for instance, by counter-attitudinal information, more negative affective responses such as anger and irritation were evoked (counter-arguing/negative thoughts were also enhanced and the source was viewed less favorable); the affective responses partially explained the stability of more important attitudes [37, 39]. These findings align with cognitive dissonance theory [44] that postulates discomfort (i.e., an affect) after encountering attitude-inconsistent information, or behaving in an attitude-inconsistent manner, especially with respect to more important attitudes.

## The present research

The present research advances the literature by integrating evidence on the impact of person-first language with insights pertaining to the predictors and consequences of attitude importance. First, aiming to replicate previous studies, we postulate that one-time exposure to outgroup descriptions that use person-first language reduces negative outgroup stereotyping (*Hypothesis 1a*) and negative outgroup affect (*Hypothesis 1b*), and increases outgroup approach behavior intentions (*Hypothesis 1c*). We also consider further outcomes, which reflect several unexplored potential benefits of person-first language [5, 19]. More precisely, we assess if one-time exposure to outgroup descriptions that employ person-first language fosters stronger positive stereotyping (*Hypothesis 1d)* as well as lower levels of dehumanization of the target outgroup (*Hypothesis 1e*).

Second, we test a boundary condition of the influence of person-first language to investigate why its effects on outgroup perceptions or behavioral intentions vary in size across different social groups—especially when comparing research that was conducted in the medical and criminal justice context. As discussed, it is conceivable that individuals attribute different levels of importance to their attitudes towards various outgroups. An attitude towards an outgroup is expected to be more important if the outgroup’s actions impact the individual’s rights or lifestyle more strongly (i.e., self-interest); if the attitude about the outgroup is more important for a relevant reference group (i.e., social identification); and if the outgroup’s activities infringe strongly on the individual’s core values (i.e., values) [38].

Using concrete examples, even if both attitudes are negative in valance, attitudes towards an outgroup that is affected by disabilities are expected to be less important than attitudes towards others who have committed a crime. A person’s disability typically does not inflict restrictions on the lifestyle of the general public. Committing a crime, on the contrary, directly and indirectly affects third parties and can have severe negative implications for the quality of life in a neighborhood. The public in the United Kingdom (UK), where the present research will be conducted, also indicated in two recent polls that ‘crime’ was one of the five most important issue facing the country [45]; the economy, health, immigration, and the environment were viewed as more important; see also Office for National Statistics (ONS, [46]). Lastly, committing a crime violates universal moral values, core values of different religious communities, as well as the law in the UK. If attitudes towards individuals who have committed a crime are attributed a higher significance than attitudes related to other people with a disability, the former should be less likely to change even if one is exposed to an intervention that aims to reduce stigmatization. Therefore, the aforementioned effects of outgroup descriptions that use person-first language (*Hypothesis 1a -1e)* are expected to be smaller for the outgroup “people who have committed a violent crime” than “people who have a physical disability” (*Hypothesis 2*).

## Method

By virtue of this being a stage-1 registered report, the data for this study is not yet available. All data, analytical scripts, and materials will be made available on this Open Science Framework registry https://osf.io/u54k8/?view_only=66cd4d8abfd94c588603abece4a2afd9 upon study completion. The departmental ethics committee of the Department of Security and Crime Science, UCL has granted written ethical approval for this study.

### Design plan

To assess the hypotheses, we will conduct a single-blind (online) experiment; participants will not know to which condition they were assigned but researchers will be aware of the respective treatments when analyzing the data. We will apply a between-subjects design that includes two factors (i.e., target group x descriptor) with two levels respectively (target group: people who have a physical disability, people who have committed a violent crime; descriptor: identity-first, person-first) as well as a control group. Simple randomization will be implemented to assign participants.

### Sampling plan

As of the date of submission of this registered protocol, the data have not yet been collected, created, or realized.

#### Data collection procedure

Participants will provide informed consent through a tick-box. Data will be collected on the online opt-in access panel Prolific Academic. Participants will self-identify by responding to an invitation on the platform and will receive reimbursement of 0.50 GBP, corresponding with the anticipated study duration of five minutes. A pre-test, which will capture attitude importance, will be conducted one week before the main study.

#### Sample size

We aim to recruit *N* = 681 participants. The sample size calculation is based on an a-priori power analysis, considering the smallest effect of interest as a reference [47]. Our goal is to obtain .95 power, with α = .01 (Bonferroni-corrected p-value for five outcomes; see below), to be able to detect also small effects (or differences) of f = .175 with univariate analyses of variance (one-way). We will only recruit participants who are UK residents and older than 18 years. We introduce the former selection criterion (i.e., being a UK resident) as we will apply definitions of disability and violent crime (see Manipulated variables–experimental stimuli) that are provided by UK institutions.

### Variables

#### Manipulated variables–experimental stimuli

In line with previous research [11, 12], the experimental conditions (and the control condition) will be defined by referring to (one of) five different groups when presenting the outcome measures (Table 1). That is, participants will complete outcome measures considering either ‘most people’ (control group), ‘violent criminals’ (identity-first language), ‘people who have committed a violent crime’ (person-first language), ‘the physically disabled’ (identity-first language), or ‘people who have a physical disability ‘ (person-first language).

**Table 1:** Instructions and group definitions.

Condition	Instructions and definition
Control group	To what extent do you agree with the following statements about most people?
negative attitudes are expected to be more important, identity-first language	To what extent do you agree with the following statements about violent criminals?
	Definition of a violent crime: harmful force is used upon a victim, with or without using a weapon, ranging from common assault to murder (CPS, 2023)
negative attitudes are expected to be more important, person-first language	To what extent do you agree with the following statements about people who have committed a violent crime?
	Definition of a violent crime: harmful force is used upon a victim, with or without using a weapon, ranging from common assault to murder (CPS, 2023)
negative attitudes are expected to be less important, identity-first language	To what extent do you agree with the following statements about the physically disabled?
	Definition of physical disability: a physical impairment that has a ‘substantial’ and ‘long-term’ negative effect on the ability to do normal daily activities (UK Equality Act 2010)
negative attitudes are expected to be less important, person-first language	To what extent do you agree with the following statements about people with a physical disability?
	Definition of physical disability: a physical impairment that has a ‘substantial’ and ‘long-term’ negative effect on the ability to do normal daily activities (UK Equality Act 2010)

#### Measured variables

Unless indicated otherwise, all measures mentioned below will apply a 5-point Likert-type scale (1 = ‘not at all’, 3 = ‘neither agree nor disagree’, 5 = ‘completely’).

*Attitude importance* will be measured in a pre-test. Attitude importance is commonly assessed by asking participants how concerned they are about a specific issue, rather than how much they care about their attitudes on this issue; both approaches have been found to be valid [15]. Participants will be presented with a list of five topics (i.e., two target and three filler topics: violent crime, taxes, physical disability, climate change, and product placement in movies) and asked to indicate their opinion about the topics (i.e., valence; 1 = very negative, 7 = very positive) as well as how important these topics are for them (1 = not at all important, 5 = very important). That is, in the pre-test, we make references neither to person- nor identity-first language.

In the main study, and after providing participants with a definition of either the term physical disability or violent crime (Table 1), we will rely on an adapted version of Enock and Over’s [48] scales that explore desirable and undesirable outgroup traits that are either uniquely human (i.e., two positive and two negative stereotypes: knowledgeable, open-minded, arrogant, controlling) or traits that humans share with animals (i.e., dehumanization: calm, curious, inflexible, unsophisticated). In doing so, we distinguish stereotyping from dehumanization [48]. Negative affect will be measured with three items (‘I would be afraid to be around ….’, ‘I would be upset if … moved into my neighbourhood’, ‘I feel disgusted by …’). Outgroup approach behavior intentions will examine willingness for intergroup contact in different settings (‘I would not want to work with …’, ‘I would not want to live near …’, ‘I would want to be friends with …’, ‘I would never be willing to date …’).

An attention check will be embedded in the list of aforementioned items (‘This is an attention check. Please answer ’Not at all’ to indicate that you pay attention.’). Lastly, participants will be asked to complete a manipulation check (‘You have just answered several questions about a specific group of people. How was this group referred to?’ Answer options: Violent criminals, People with a physical disability, Most people, The physically disabled, People who have committed a violent crime, My friends) and indicate whether they themselves have ever committed a violent crime (No/Yes) or describe themselves as a person with a physical disability (No/Yes).

#### Indices

The following indices will be created by calculating mean scores: positive and negative traits that are uniquely human (positive and negative stereotypes; two items each) or shared with animals (dehumanization; four items); negative affect (three items); as well as willingness for intergroup contact (four items).

### Analysis plan

#### Data exclusion

We will exclude data from all participants who did not fully complete the study, failed either the attention or the manipulation check, or who indicated that they themselves had ever committed a violent crime or describe themselves as a person with a physical disability.

#### Missing data

All questions will require responses, such that no missing data will occur. Incomplete responses will be removed (i.e., listwise deletion).

#### Confirmatory analyses

We will test for median absolute deviation to detect potential univariate outliers for all outcome variables [49]. We do not plan to exclude outliers but will report their presence. Mean scores and standard variations of as well as bi-variate correlations between all measures will be calculated.

Examining Hypothesis 1a-e (i.e., the effects of person-first language), we will test the assumptions for univariate analysis of variances (ANOVA) for all five outcome measures separately for each of the two descriptor pairings (see Huang [50] who suggests multiple ANOVAs with p-values that are corrected for multiple comparisons rather than a multivariate analysis of variance). Once assumption checks confirmed the suitability of the tests, we will conduct five two-tailed ANOVAs, comparing the indices of positive and negative stereotyping, dehumanization, negative affect, and approach behavior intentions between the identity-first, person-first, and control condition regarding the descriptor pairing “people with a physical disability/the physically disabled”. The analyses will then be repeated for the descriptor pairing “people who have committed a violent crime/violent criminals”. Planned contrasts will be used to investigate for each descriptor pairing whether average negative outgroup perceptions and affect are lower, positive perceptions and approach behavior intentions are higher in the person-first as compared to the identity-first language condition. It is further expected that negative outgroup perceptions and affect are lowest as well as positive perceptions and approach behavior intentions are highest in the control condition. Effect sizes and confidence intervals will be computed. To correct for the multiple ANOVAs in each descriptor pairing, p-values are adjusted using the Bonferroni correction. We address concerns about the reduced power of the tests by using the corrected p-value of p = .01 (i.e., .05 divided by five) in the a-priori power analysis (see above).

To investigate Hypothesis 2, we will first verify that participants did, indeed, hold largely negative views on the topics of violent crime and physical disability. Given the scale mid-point of 4, we will conduct a one-tailed one-sample t-test with the test value 3, assessing whether the mean of the two items is below 3. We will conduct a two-tailed paired t-test to confirm the speculation that attitudes towards violent crime are more important than those towards physical disability. Both aforementioned analyses will include the whole sample.

Should we not identify a significant difference in attitude importance, no further analysis will be conducted. If attitudes towards violent crime are considered more important, we will then conduct two-sided equivalence tests for each of the six outcome variables in the sub-sample that expressed views on an outgroup for which negative attitudes are expected to be more important (i.e., violent criminals/people who have committed a violent crime). The effect sizes attained in the aforementioned ANOVAs (i.e., partial eta square that is converted into Cohen’s d) for the sub-sample that reported views about people who have a physical disability/the physically disabled will be used to define the lower and upper bounds of the equivalence tests, in other words, the smallest effect size of interest [51]. In doing so, we will assess whether we can reject the null hypothesis that the mean differences that were identified are at least as small as the lower or at least as large as the specified upper bound.

### Timeline

We have already received ethics approval for the study (see attachment). The study is also already programmed in Qualtrics. We intend to commence data collection immediately after the Stage-1 registered protocol is accepted, allowing one week to implement requested changes, and expect to have received all data within two weeks. Data processing and analysis will be completed within three weeks, and we intend to complete the full report within three months of having received approval of the Stage-1 proposal.
